# Investigating correlations between illness and defensive behaviour approach: A case of twin cities of Pakistan

**DOI:** 10.1016/j.heliyon.2021.e07327

**Published:** 2021-06-16

**Authors:** Tanzila Akmal, Faisal Jamil

**Affiliations:** School of Social Sciences and Humanities, National University of Sciences and Technology (NUST), Islamabad, Pakistan

**Keywords:** Waste management, Health production function, Defensive behavior

## Abstract

Municipal solid waste (MSW) management has emerged as a major problem for modern societies in recent decades. An optimal waste management system is essential to prevent the pollution burden and associated health related issues. This study carries out an empirical evaluation of the illness caused by inadequate solid waste management in the metropolitan of Rawalpindi-Islamabad. The model is based on utility-maximizing consumer behavior and predicted probability of disease in the household is estimated by employing “seemingly uncorrelated bivariate probit model”. Primary data obtained through multistage random sampling that comprises of 849 respondents. The findings show that irregular waste disposal sites in the vicinity of residences cause illness. The key findings indicate that distance from dumpsites and use of contaminated water adversely affect the health outcomes. Furthermore, the results show that respondents were unable to engage in defensive activities due to a lack of awareness. Oft-times, the waste is dumped in illegal sites that is burnt thus causing excessive air and ground water pollution. The results shed light on the respondents' understanding of the negative consequences of excessive waste disposal and study suggests measures that motivate households to engage in defensive activities through effective campaigns and capacity building programmes that ensure sustainable solid waste management.

## Highlights

• An overview of the household solid waste generation and management practices.
• Construct households' illness function regarding improper waste disposal practices.
• Construct households' defensive behaviour regarding illness function.

## 1 Introduction

Increased economic growth in the twenty-first century has resulted from the industrial civilization that has transformed countries all over the world. United Nations Department of Economic and Social Affairs reported that, the world's urban population was 55% of the total population in 2018, but is estimated to continue rise up to 68 percent by 2050 and more than 90% of population growth have taken place in Asia and Africa [1]. The growth of industrial society combined with population growth around the world, considerably contribute to the increase in the volume and variety of waste. However, if not adequately handled, municipal solid waste (MSW) causes negative externalities [1, 2, 3].

Municipal or household wastes are often produced from a variety of sources, depending on the human and industrial activities. Most of municipal solid waste is produced from households (55–80%) followed by commercial or market areas (10–30%), varying in amount from streets, industries, and institutions [3, 4]. Waste from different source is heterogeneous in nature such as organic waste, yard waste, wood, plastics, papers, metals, leather, rubbers, batteries, paint containers, textiles, construction and demolishing materials etc. [2, 3]. Due to microbial decomposition, atmospheric conditions, refuse characteristics, and land-filling activities, waste disposal creates harmful gases and leachates which causes plenty of environmental and health problems. Improper disposal of waste also includes, sharp objects such as syringes, razors and blades can directly pose serious health hazards. Local governments in developing countries are unable to collect and separate waste from all potential waste producers due to a lack of operational capacity and difficulties in recovering waste management costs [2, 3, 4, 5].

In Pakistan, Municipal Solid waste (MSW) management is consists of door to door and container based collection system. In most Pakistani cities, only 60% of the waste produced is collected, and more than 90% of collected waste is dumped openly. The uncollected waste can be noticed in empty plots, drains, and open sewers, along streets, roads and railway lines. The amount of solid waste produced in Pakistani cities is estimated to be 55,000 tons per day [6, 7]. A shortage of skilled manpower, inadequate policy and institutional support, insufficient technical and financial resources are the major obstacles to proper solid waste management (SWM) in Pakistan [8, 9].

Rawalpindi-Islamabad are rare examples of twin cities that are geographically similar to each other and ultimately converge. Despite certain similarities due to their origins and cultural belief systems, the so-called twin cities are far from identical. Local governments are failed to adequately handle waste in Islamabad-Rawalpindi. Twin cities do not yet have a comprehensive solid waste management plan, nor does it have sanitary landfill. Open landfilling have been practiced by municipal management and private waste handling authorities, which is posing a significant challenge to public health and environmental quality. In additional, open dumping acts as breeding ground for many disease vector, such as malaria, typhoid fever, diarrhea and other infections and toxic gases, air, water and land pollution. Uncollected waste heaps are almost everywhere [8, 9, 22].

The majority of public and private agencies in the twin cities use waste collection and disposal approaches that are radically different from operationally available best practices for sustainability and circular economy. Therefore, local governments and private service provider have been unable to resolve the problem of solid waste management [9]. The street side and empty plots are loaded with waste in major cities of Pakistan's such as Lahore, Islamabad and Karachi. Thus, dumping uncollected and unauthorized waste causing destruction of the ecosystem [10, 11].

Health is a key component of inclusive development, both as a major dimension of wellbeing in itself, and because of its links to income, jobs, and other aspects of well-being. According to the Pakistan National Conservation Strategy, water-related diseases account for 40% of all communicable diseases. One of the main causes of waterborne diseases is the addition of municipal sewage and industrial wastewater to drinking water [12]. According to International Union on Conservation of Nature (IUCN) report (2007) infant deaths caused by water-related diarrhea are 60% of total death rate in Pakistan, the highest ratio in Asia. In additional, Pakistan has the world's highest infant mortality rate (12.6%) and fertility rate (7%), indicating the country's poor health.

The consumption and waste disposal practices of households have a significant effect on the environment [13]. Social and consumption behavior of household are imperative factors that contribute to waste generation. Environmental awareness affects household social and consumption behavior. As a result, environmental awareness led to defensive actions, which is needed to prevent the negative effects of solid waste [14]. Therefore, Health and environment are inseparable elements of development that cannot be sustained independently [14, 15].

The influence of waste disposal on health is consistent with the idea that households should engage in defensive measures by evaluating the severity of adverse effects [16, 17, 18, 19, 20, 21]. Household defensive behavior is affected by environmental awareness, time, money and these factors serve as a barrier to defensive behavior [21, 22, 23, 24]. On the basis of above literature, we develop the following hypotheses:

H1 Unsafe waste disposal has effect on prevalence of diseases among households.

H2 Defensive behavior is endogenous to health production function.

First, to the author knowledge, there has been little research on this topic in recent years, especially in developing countries like Pakistan. Limited evidences are reported on association between household waste management and health effects. As a result, the aim of this research was to see if there was a correlation between dumpsite exposure and the health of nearby residents. The health production function approach is used in the analysis to conduct an objective evaluation of the health damages based on distance from the dumpsite. We'd like to see if there's any epidemiological evidence that waste disposal sites have negative impact on public health. The research findings will provide information to the target authorities so that they can make decisions about waste management and determine the contours of a comprehensive and feasible strategy to address issues and, as a result, optimize health indicators for metropolitan of Islamabad- Rawalpindi.

Rest of the paper is as follows. Section 2 outlines the theoretical framework of the study. Section 3 outlines empirical model, sampling process and survey design. Section 4 presents analysis of relevant variables and discusses the results. Finally, Section 5 concludes the study.

## 2 Theoretical framework

The sensitivity analysis focuses on changes in human actions to see how a shift in an environmental resource affects people's health. A model for estimating losses from improper disposal of waste based on the theory of utility maximizing consumer behavior has been evolved for determining the probability of illness for a household. The choice of waste disposal and the health of households vary greatly and is contingent on their choice of residential location. The choice of location can be divided into two categories: residences near a dumpsite and residences away from a dumpsite. Negative externalities are imposed on households living near a dumpsite or upstream by households living away from the dumpsite. We assume a utility-maximizing household that derives utility from consumption of composite good, waste disposal, and health [25, 26, 27]. The utility function takes the following form.

(1) $$U=f\left(C_{i},G_{i},\;H_{i}\right)$$

where (*C*_*i*_) represents expenditures on non-health commodities, (*H*_*i*_) is the state of being healthy and ($G_{i}$) is the waste that needs to be disposed of. Utility is an increasing function of consumption of composite good (*C*_*i*_) health (*H*_*i*_) and decreasing function of garbage ($G_{i}$). The first- and second-order conditions are of the following form.

$$U_{\overset{⌢}{C}}U_{\overset{⌢}{H}}>0;\;U_{\overset{⌢}{G}}<0$$

$$U_{C\overset{⌢}{C}}U_{H\overset{⌢}{H}}\;U_{G\overset{⌢}{G}}<0$$

Waste without treatment produces larger negative externalities in term of human health and environment. The study introduces a health production function in order to study more clearly the impact of negative externality. To express health status, we specify health production function following [25, 26, 27]. Household's health status is modelled as a function of the waste disposal and defensive behavior to minimize the probability of being ill. Thus, the “health production function” can be defined as follows.

(2) $$H_{i}=f\;\left(G_{i}\;,D_{i};\;Z_{i}\right)$$

where $H_{i}$ represents the health status of an individual. $G_{i}$ is the garbage.^1^ In Eq. (2)$\;D_{i}$ represents the defensive activities of households to reduce the likelihood of being sick; Zi represents household characteristics. Other characteristics of a household can comprise of income, education, employment status, household size etc. Household maximizes its utility given the following budget constraint.

(3) $$Y_{i}=A_{i}+W_{i}\left(T_{i}-L_{i}\right)=P_{c}C_{i}+P_{d}D_{i}+P_{g}G_{i}$$

Thus, $Y_{i}=P_{c}C_{i}+P_{d}D_{i}+P_{g}G_{i}$.

For optimization, following Lagrange multiplier is solved.^2^

(4) $$L={C_{i}}^{\alpha }{G_{i}}^{\beta +\gamma \delta }{D_{i}}^{\delta \vartheta }-\lambda \left(Y_{i}-\;P_{c}C_{i}+P_{d}D_{i}+P_{g}G_{i}\right)$$

(5) $${G_{i}}^{*}=\frac{\left(\beta +\gamma \delta \right)}{\alpha +\beta +\gamma \delta }*\frac{\left(Y-P_{d}D_{i}\right)}{P_{g}}$$

Eq. (5) describes the household demand for waste disposal. Household is allocating his/her income for consuming different goods, such as waste disposal and defensive activities. There is negative relationship between waste disposal and cost of waste disposal activities whereas, a positive association between income, and other defensive activities such as safe waste disposal, use of treated water etc.

(6) $${D_{i}}^{*}=\frac{\delta \vartheta }{\alpha +\delta \vartheta }*\frac{\left(Y-P_{g}G_{i}\right)}{P_{d}}$$

Eq. (6) is the demand for defensive activities, where income, prices, and waste disposal are as argument in demand function. Demand function for defensive behavior shows how much a household is allocating its resources to defensive activities. By putting both demand functions into health production function, we get, illness/health funcation of household, which has to be estimated empirically.

(7) $${H_{i}}^{*}={\left(\frac{\left(\beta +\gamma \delta \right)}{\alpha +\beta +\gamma \delta }*\frac{\left(Y-P_{d}D_{i}\right)}{P_{g}}\right)}^{\delta }{\left(\frac{\delta \vartheta }{\alpha +\delta \vartheta }*\frac{\left(Y-P_{g}G_{i}\right)}{P_{d}}\right)}^{\delta }$$

Eq. (7) is the health demand function. The level of health and the price of various goods are negatively correlated. If the price of commodity (${G_{i}}^{*}$and ${D_{i}}^{*}$) increases, it worsens the level of health and vice versa. In case of less developed countries, it is presumed that waste is improperly collected and disposed of by management authorities, causing serious health problems to the people living near the dumpsites. This equation helps us to estimate difference in the health status of heterogeneous households.

## 3 Econometric specification of the model

The aim of the study is to quantify the health damages associated with the improper disposal. Therefore, both the functions namely; health production and demand for defensive activities functions are being evaluated by using seemingly unrelated bivariate probit models^3^ and empirical model is derived from the study conducted by (Dasgupta, 2004) on health damages from contaminated water [25]. The estimation process includes estimating the relationship between disease and exposure to pollution while controlling other factors that influence the health function of households. The model will then be estimated by using binary data set at household-level.

High exposure to pollution is associated with higher chances of being sick that in turn, leads to more defensive activities. Subsequently, health and defensive activities are highly interlinked allowing for joint assessment of health and defensive activities at the household level. There are some explanatory variables that simultaneously influence the health and defensive behavior, the corresponding error terms are subject to contemporaneous correlation. This correlation cannot be identified if both equations are estimated independently. Since, both equations are “seemingly” unrelated rather than independent. Under these conditions, the calculation of two independent equations may lead to consistent yet inefficient coefficient values.

We therefore, assumed that defensive activities^4^ equation comprised both observable and unobservable variables; observable variables ($x_{1})$ are income, education, and defensive activities and unobservable factors ($Z^{*}$) are contaminated water, illegal dumpsite, waste burning, and dumping into *nala* etc.

By summarizing both observable and unobservable factors into vector form, equation for defensive behavior is written as follows. Where $D^{*}$ is latent variable and used in dichotomous outcome.

(1) $$D^{*}=x_{1}\alpha_{1}+\beta_{1}Z^{*}+\varepsilon$$

Since, the risk factors are unobservable, so they will be absorbed into the error terms. We get the following equation by spearing out the known risk factors and the error term from Eq (1’).

(2) $$\nu_{1}=\;\beta_{1}Z^{*}+\varepsilon$$

So Eq (1’) can be written as

(3) $$D^{*}=x_{1}\alpha_{1}+\nu_{1}$$

In Eq. (3’) $x_{1}$ is independent of $\nu_{1}$ and get consistent estimators by regressing defensive behavior of the household on selected explanatory variables.

A binary specification is assumed for the second reduced-form equation regarding to health^5^ and its associated determinants. From theoretical model we know that health is the function of defensive activate. Thus, bases on theoretical model health equation is constructed, where latent variable $H^{*}$ is defined as follows.

(4) $$H^{*}=x_{2}\alpha_{2}+\beta_{2}Z^{*}+\gamma D^{*}+\mu$$

Here $x_{2}$ is also a set of households' attributes. Again, unobservable factors *Z*∗ include the contributing factors of illness such as presence of rodents/insects, and irregular dumpsite etc. Health can be improved by adopting defensive behaviour, so coefficient γ is assumed to be positive.

(5) $$\nu_{2}=\;\beta_{2}Z^{*}+\mu$$

So, Eq (4’) can be written as

(6) $$H^{*}=x_{2}\alpha_{2}+\gamma D^{*}+\nu_{2}$$

Although, error terms ε and μ are considered to be independent of each other, but estimation of (4’) yields inconsistent estimators because hidden risk factors introduce correlation between D∗ and $\nu_{2}$ in health equation. To get consistent estimators, $\nu_{2}$ should be independent to D∗.

Therefore, by substituting (1′) into (4’) and we obtain a second reduced form equation, which is independent of defensive behaviour. It only depends on household personal characteristics unobservable risks and illness related to waste. Thus, second reduce form equation can be given as follow:

(7) $$H^{*}=x_{2}\alpha_{2}+x_{1}\left(\text{γ}\alpha_{1}\right)+\left[\left(\text{γ}\beta_{1}+\beta_{2}\right)Z^{*}+\left(\text{γ}\varepsilon +\mu \;\right)\right]$$

The error term of (7′) is correlated with the error term of the first equation, $v_{1}=\beta_{1}Z^{*}+\varepsilon$. As a result, the non-independence of the likelihood of defensive action (1′) and health status (7’) can be estimated jointly as simultaneous equations by using “seemingly unrelated bivariate Probit model” (see Table 1).

**Table 1:** List of variables.

Variable Codes	Variable Types	Variables Measurement	Expected outcomes
Waste related illness	Dummy	Being ill = 1; otherwise 0	
Partial segregation	Dummy	If yes = 1; otherwise 0	
Income	Continuous	The log of monthly income of the household head	+sig
Distance from dumpsite	Dummy	4 dummy variables are considered according to distance from dumpsites. Respondents living away from dumpsite (≥ 500m) are treated as omitted category.	+sig
Prevalence irregular dumpsite	Dummy	If yes = 1 otherwise 0	+sig
Burn waste	Dummy	If yes = 1; otherwise = 0	+sig
Waste throw into nala's	Dummy	If yes = 1; otherwise = 0	+sig
Education	Dummy	4 dummy variables are considered according to education level. Uneducated respondents are treated as omitted category.	± sig
Access to collection services	Dummy	If yes = 1; otherwise = 0	+sig
Source of water	Dummy	If tap water = 1 otherwise 0	± sig
Use of treated water	Dummy	If yes = 1; otherwise 0	+ sig
Use of contaminated water	Dummy	If yes = 1; otherwise 0	- sig
Water supply line	Dummy	If water supply line passing through sewerage line = 1; otherwise 0	± sig
Presence of rodents	Dummy	If yes = 1; otherwise = 0	-sig

### 3.1 Study design and sample size

The study employs a cross-sectional quantitative dataset obtained from the residents of the selected localities of the twin cities of Rawalpindi and Islamabad through structured questionnaire. The questionnaire is self-administered and paraphrased into Urdu language as most of the respondents would not be able to answer in English. The questionnaire is based on literature review and with consultation of the sectoral experts before and after pretesting.

The study assumes that waste is dumped at a particular location in each community. Households were classified into two categories based on their choice of residence: those living near the dumpsite (within ≤ 100m to ≥ 500m radius) and those living away from the dumpsite (≥500 m). The most common activity in poor neighborhoods is open dumping in vacant plots and alongside sewage streams (Nalas). In poor communities, solid waste collection is not recognized as the primary component of service delivery. The questionnaire includes about eight recorded toxic exposure symptom variables across households, including (1) diarrhea; (2) malaria; (3) dengue; (4) asthma; (5) skin problems/irritations; (6) cholera; (7) typhoid and (8) fatigue.

**Figure 1:**
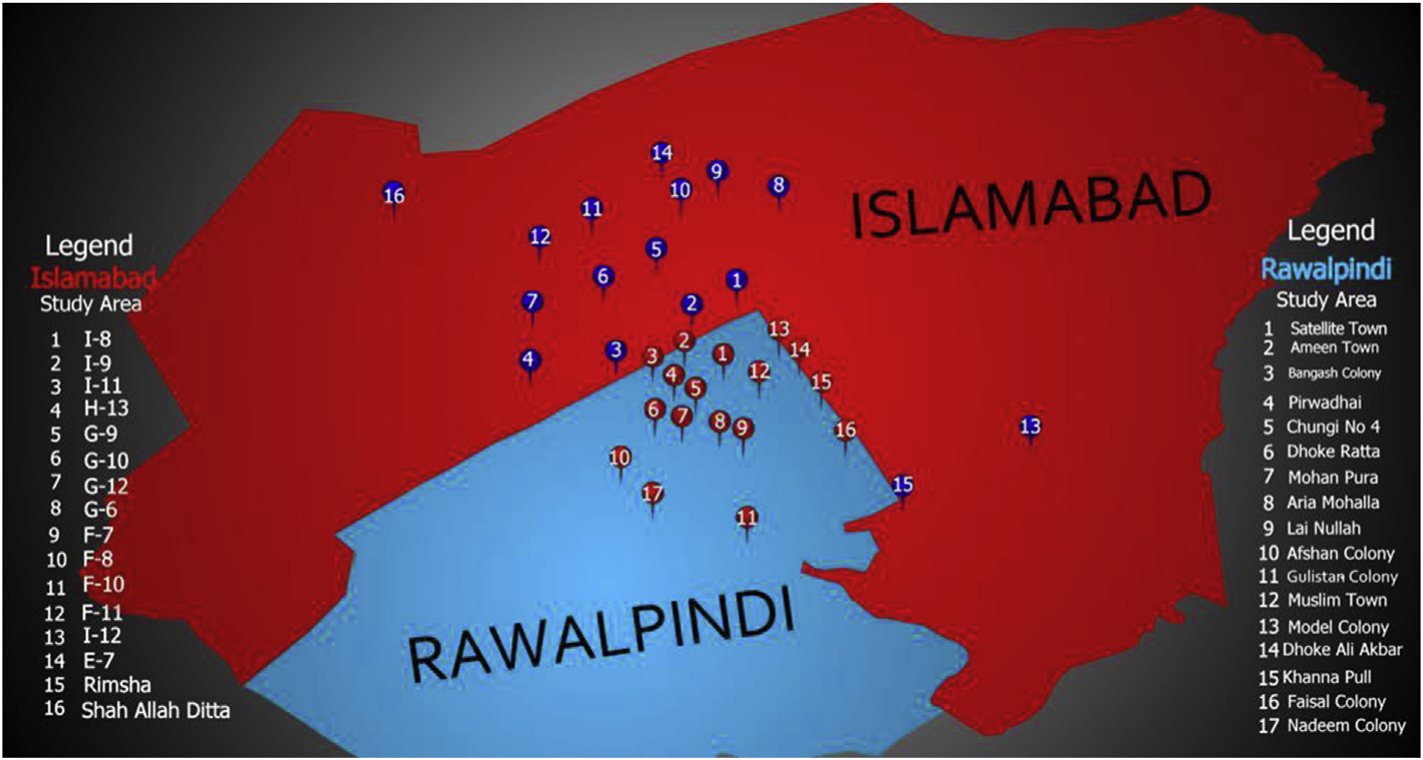
Map of the twin cities and locus of sampling clusters.

For data collection, multistage random sampling is used. In the first stage, we chose residential areas at random. In the second stage, streets and houses inside streets were selected at random in a subject area. The survey is divided into 35 sectors and towns at the third stage, with 24 households from each sector chosen for interviews. Selected sites are further subdivided into 17 nearby dumpsites and 18 residential areas away from dumpsites. A total of 849 households were interviewed at the final step (July–August 2019). Figure 1 gives a glimpse of the survey site and properly numbered sampling basis.

## 4 Results and discussions

Demographic statements that were incorporated in the survey include household income and respondents’ gender, age and education. Majority of (65%) of respondents in the sample were female as the survey was conducted in the day time when male household members were on their job and mostly housewives were interviewed as given in Appendix Table 1. Education level of respondent is rather inconsistent. A similar data trend is exhibited for monthly family income where the lowest and the highest income groups have the highest frequencies as can be seen in the table.

### 4.1 Waste generation and composition

Biodegradable waste (primarily kitchen waste) accounts for the majority (45.5%) of household solid waste (HSW) production (Appendix Table 2). These results are in line with those of many other developing countries, including Nigeria [28, 29]. According to ESCAP and UN-Habitat Pakistan [30], twin cities are producing growing amount of solid waste, which has increased from around 500–600 tons per day in 2004 to around 800–1,000 tons per day in 2011. According to the survey data [8], waste generation in twin cities is 1852kg per day.

According to the survey findings, the majority of respondents (44.5%) generate organic waste weighing between (0.5–1.75) kg/day, which must be disposed of. This finding is consistent with previous research conducted in Beijing, China (0.8 kg/cap/day), Ambon, Indonesia (0.9 kg/cap/day), and Lahore, Pakistan (0.84 kg/cap/day) [31]. Just 1.7 percent of respondents generate 5–6.25 kg of waste every day. A household's waste generation can be affected by a variety of factors, including family size, education level, and monthly income [32].

### 4.2 Household disease burden

**Figure 2:**
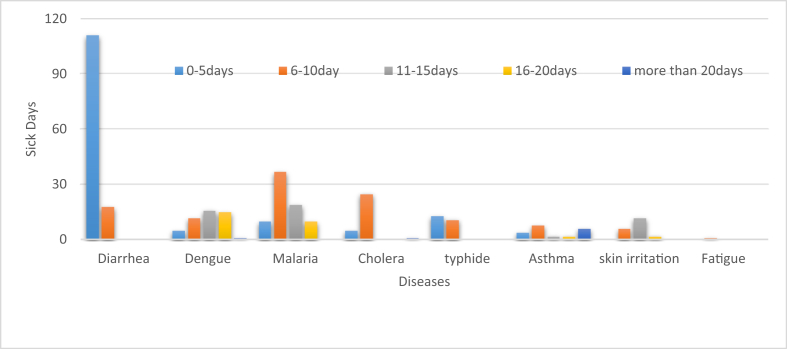
Household disease burden [8].

Figure 2 depicted that respondents who was sick for a long time due to malaria or dengue fever corresponding to their exposure to the household hazardous waste (HHW)^6^. 111 respondents reported having diarrhea over a period of 0 to 5 days, and 25 respondents had cholera for a week or more.

### 4.3 Size and age of irregular dumpsites

**Figure 3:**
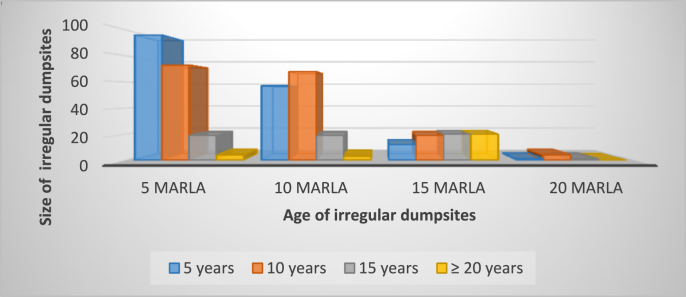
Age and size of dumpsite.

**Figure 4:**
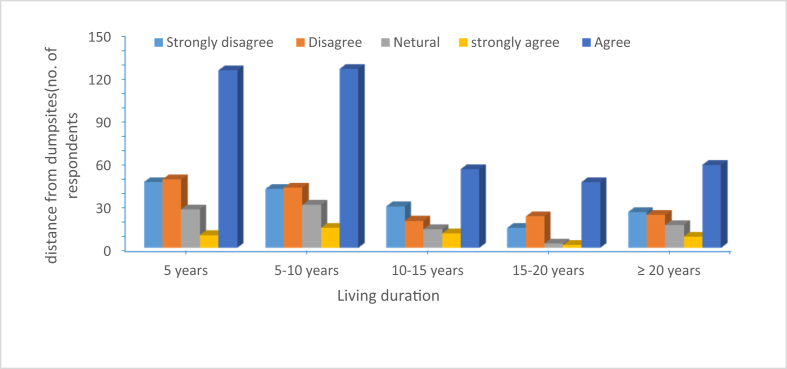
Distance and living duration near dumpsites.

Study data (Figure 3) revealed that majority of respondents were living near dumpsites since (5–10 years). Number of studies shows that with the passage of time and seasonal variation, solid waste deteriorated over time increased, as a result, seasonal and age changes have a major influence on leachate composition. Furthermore, studies have shown that even after a landfill site is closed, a mixture of physical, chemical, and microbial processes in the waste can continue to generate polluted leachate, and this process could last for 30–50 years [55, 56] (see Figure 4).

### 4.4 Living duration and distance from dumpsite

Figure 4 indicates the respondents acceptance of the dump site. The findings show that 249 participants living near and who have lived from less than 5 years–10 years were seriously concerned to the disposal sites located closer to their homes. whereas 158 respondents living away from dumpsites was fine with presence of dumpsite. In addtion results indicated that respondendts have lack of awareness of the long-term risk associated with waste disposal.

### 4.5 Results from the econometric estimation

The dependent variable for health equation, being sick as a result of a waste-related disease and the independent variables were the source of water, supply line^7^, presence of rodents, use of contaminated water^8^, the presence of irregular and illegal dumpsites^9^, waste burning^10^, waste dumped into *Nalas*, distance from dumpsite and the educational level of household head.

The dependent variable is partial segregation of waste–partially segregation of waste is the only option for averting behavior practiced by the surveyed households. Explanatory variables considered for the defensive behavior are income, and treated water from any source, access to waste collection services, irregular dumpsite, distance from dumpsite, burning, waste throw into nala's and education of head. Income of household was included in logarithmic form and is self-explanatory. The educational background is a categorical variable, which was defined as five dummies each representing a certain level of educational attainment. Dummy 1 (the omitted category) takes a value of 1 if the person is uneducated otherwise zero, dummy 2 takes a value of 1 if the household has completed his/her primary education, dummy 3 is for those who have completed their secondary education, dummy 4 who have completed their higher education and dummy 5 who have completed their professional/formal education. Distance from dumpsite is also categorical variable, which was defined as five dummies each representing a certain level of distance from disposal site [25]. Dummy 5 (the omitted category) takes a value of 1 if the distance ≥500 otherwise zero, dummy 4 takes a value of 1 if the distance is between 301m - 400m, dummy 3 takes value 1 if the distance is between 201m - 300m otherwise zero, dummy 2 takes value 1 if the distance is between 101m - 200m otherwise zero and dummy 1 if the distance is within 100 m [5,26]. The precise results from the specification that was finally selected for further analysis are given in (Table 2). The results are briefly summarized below.

**Table 2:** Bivariate probit results.

Equation 1: Dependent variable = Waste related illness			
Independent variable	Coefficient	St. errors	P- values
Presence of rodents/insects	-0.1602	0.1812	0.377
Dummy for distance_1	1.3656∗∗∗	0.1950	0.000
Dummy for distance_2	1.3141∗∗∗	0.1915	0.000
Dummy for distance_3	1.1066∗∗∗	0.2118	0.000
Dummy for distance_4	1.3909∗∗∗	0.3424	0.000
Irregular dumpsite	0.3165∗	0.1834	0.084
Source of water	0.1181	0.1495	0.430
Use of contaminated water	0.4690∗∗∗	0.1229	0.000
Education of head_level2	-0.2458∗	0.1488	0.099
Education of head_level3	-0.1686	0.1637	0.303
Education of head_level4	-0.0167	0.1869	0.929
Education of head_level5	-0.1951	0.1853	0.292
Supply line	-0.1091	0.1125	0.332
Burning	-0.1493	0.3276	0.649
Waste throw into nala's	-0.1977.	0. 1281	0.123
Constant	0.1157	0.1766	0.513
**Equation 2: dependent variable= partial segregation of waste**			
Burning	-0.7101∗	0.4043	0.079
Waste throw into nala's	0.4653∗∗∗	0.1408	0.001
Irregular dumpsite	-0.7321∗∗∗	0.1267	0.000
access to waste collection services	0.7515∗∗∗	0.1192	0.000
Use of treated water	0.6679∗∗∗	0.0794	0.000
Education of head_level2	0.0047	0.1584	0.976
Education of head_level3	0.2997∗	0.1708	0.079
Education of head_level4	0.3185∗	0.1926	0.098
Education of head_level5	0.4025∗∗	0.2003	0.045
Log (income)	1.8862∗∗∗	0.2072	0.000
Dummy for distance_1	-0.7329∗∗∗	0.1709	0.000
Dummy for distance_2	-0.9917∗∗∗	0.1781	0.000
Dummy for distance_3	-0.6912∗∗∗	0.2015	0.000
Dummy for distance_4	-0.5873∗∗∗	0.2527	0.000
Constant	-9.2794∗∗∗	1.0311	0.000

**Notes:** Number of observations used is 849. “∗∗∗ indicates t-statistic is acceptable at the 99 per cent level of confidence, ∗ indicates acceptance at the 90 per cent level of confidence and ∗∗ indicate 95% level of confidence”.

To begin, the Wald chi^2^ statistic reveals that the model specification has strong explanatory power. Since this is a probit exercise, the variable coefficients cannot be interpreted explicitly in terms of their magnitudes. However, signs and significance levels are worth examining. The null hypothesis is rejected by the probability ratio test of *ρ* = 0, implying that the specification is appropriate; and it is accurate to structure both equations together as a “seemingly unrelated bivariate probit”.

In Eq. (1), set of explanatory variables are used to model the waste related illness. Our study revealed that distance from dumpsite is important determinant of incidence of illness. Residents living near the dumpsite had a significantly higher risk of having diarrhea, cholera, dengue, malaria and asthma compared to residents living away from dumpsite. A number of community health studies that examined a wide variety of health issues linked to environmental exposure to a landfill supported these results [33, 34, 35, 36, 37, 38, 39, 40, 41, 42, 45]. The use of contaminated drinking water has a and significant positive relationship with illness, implying that households that rely on tap water are more likely to suffer from water-related diseases including diarrhea and cholera. This result implies that water is contaminated and the fact that water supply lines in poor communities pass through drainage system [43, 50]. Groundwater contamination caused by improper waste disposal within sectors and water bodies (such as Nalah Lai) has emerged as a critical issue for policymakers and planners. Results are consistent with [39, 48, 50]. A research in India compared the hydro-chemical natures of landfills to assess their effect on solid water, leachate, and groundwater [43, 44, 46]. The findings revealed that the samples had a high concentration of heavy metals.

Other risk factors that are potentially important in explaining waste-related diseases have been discovered to play a significant role. Among other aspects, irregular waste disposal is significantly determining the incidence of being sick [25, 26]. This finding is also significant in the debate over the relative strength of municipal service providers in planning and assessing measures to provide adequate and fully effective waste collection services. Waste-related illness does not appear to be primarily driven by burning, dumping waste into Nalas, or water supply lines passing through drainage. Furthermore, the education level of household head is irrelevant as a predictor of illness and is inversely related to the likelihood of illness [25, 47].

Eq. (2), demonstrates that household income is significant and positive determinant of defensive behavior. High-income households are in a better position to purchase information and have a greater capacity to make alternative choices that affect their health, such as improved hygiene, living conditions [25, 43, 46]. Moreover, the decision to engage in defensive behavior is influenced by illiteracy. A higher level of education for the household head is associated with a higher likelihood of defensive behavior [44]. Disposal of waste into *Nalas* act as an important proxy risk factor for potential water contamination and encouraging defensive behaviour [45, 48, 49]. It is difficult to characterize the outcome in terms of waste burning and the existence of irregular dumpsites since these variables capture some undefinable aspects of the socio-economic levels of households. Results indicate that both burning waste in backyard and presence of irregular dumpsite has neagative impact on defensive behavior. The results are identical to what was found during the field survey. People lack the awareness and do not accept that excessive waste disposal is the source of their diseases [8, 57] From the residents' perspective, dust, noise, and groundwater effects are not big concerns [44]. Furthermore, access to waste collection facilities and water purification facility such as, bottle water, filtered water, boiling and chlorination is significantly supported the defensive behavior of households [50, 51, 52, 53, 54, 55, 56].

## 5 Conclusions and policy implications

The research on effects of waste disposal site on human health and the environmental quality has evoked mixed reactions from academics, that makes it a dynamic analysis. The aim of this study is to know about the illness function of households in the Islamabad-Rawalpindi metropolitan area of Pakistan. To better understand the existing health issues associated with the dumpsite, questionnaire-based interviews were performed. This study looked at the health effects of the irregular disposal sites in their vicinity based on the distance from the dumpsite. The demographic, geographical and socio-economic factors that impact public health are defined through seemingly uncorrelated bivariate probit regression analysis.

The results confirmed a relationship between living close to dumpsite and damage to the respiratory system. The majority of residents, both nearby and far away, reported that the dumpsite is a breeding ground for disease vectors, causes diseases, and makes the environment filthy. Residents who lived near (in range 100m – 400m) a municipal waste disposal site, showed an association between proximity to dumpsite and different diseases such as dengue, malaria, asthma, diarrhea and skin issues. The health risk associated with groundwater contamination in this study demonstrates the critical importance of sanitary landfills. Additionally, it acts as a signal of the state's responsibility to provide better health for its residents by ensuring that important amenities, particularly essential ones like clean and safe water availability, removal of disposal sites within residential areas. The study concludes that the dumpsite should be adequately maintained in order to minimize its environmental and health impact. In additional, to prevent health and environmental hazards, landfills should be built far away from urban areas and institutions and municipality authorities should pay attention on removal of irregular dumpsite from residential areas. The findings and discussion in this paper are expected to create lucrative prospective to local municipalities and all stakeholders. The findings are critical for improved urban waste management and campaigns to raise public awareness of the adverse effects of dumping sites. The general public can play an important role in reducing the negative impact of dumpsites by improving their defensive behavior.

## Declarations

### Author contribution statement

Tanzila Akmal: Conceived and designed the experiments; Performed the experiments; Analyzed and interpreted the data; Contributed reagents, materials, analysis tools or data; Wrote the paper.

Faisal Jamil: Contributed reagents, materials, analysis tools or data.

### Funding statement

This research did not receive any specific grant from funding agencies in the public, commercial, or not-for-profit sectors.

### Data availability statement

Data will be made available on request.

### Declaration of interests statement

The authors declare no conflict of interest.

### Additional information

No additional information is available for this paper.

## Figures and Tables

**Table 1:** Social-economic information of respondent.

Gender	Age				
	15–20	21–30	31–40	41–50	>51
Female	18%	26%	14%	6%	1%
Male	11%	11%	13%	0.4%	0.1%
Education	Income				
	<30k	31k-50k	51k-70k	51k-70k	71k-1-lac

Illiterate	11.0%	6.8%	2.5%	0.8%	3.1%
Primary	3.6%	1.2%	1.1%	0.4%	0.6%
Secondary	8.3%	4.9%	4.5%	1.5%	5.5%
Higher	3.5%	4.5%	5.5%	2.3%	6.7%
Professional	3.2%	3.8%	3.3%	2.9%	8.7%

**Table 2:** Waste composition.

Index	Item	Waste measurement	Frequency	Percentages
Waste composition	organic	0.5–1.75 kg	379	44.5
	plastic	2–2.75 kg	259	30.4
	Glass	3–3.75 kg	142	16.7
	Paper	4–4.75 kg	55	6.4
	other	5–6.25 kg	15	1.7

## Footnotes

1 Garbage is disposed either by burning or open dumping; both have associated health risks and environmental wellbeing costs such as smoke, odor, or any other form of air and water pollution (Theoretical and empirical model is derived from DASGUPTA, 2004).

2 We are assuming Cobb-Douglas utility function and Cobb-Douglas health production function respectively $U\left(C,Wd,H\right)=\;{C_{i}}^{\alpha }{G_{i}}^{\beta }{H_{i}}^{\delta }$ And $H\left(Wd,D;Z\right)={G_{i}}^{\gamma }{D_{i}}^{\vartheta };{Z_{i}}^{\psi }$. By substituting health production function into utility function, we get $U={C_{i}}^{\alpha }{G_{i}}^{\beta +\gamma \delta }{D_{i}}^{\delta \vartheta }$.

3 The bivariate probit model is a joint model for two binary dependent variables whose disturbances are assumed to be correlated. It generalizes the index function model from one latent variable to two latent variables that may be correlated.

4 Defensive activity is dependent variable in this analysis. Since segregation is rare in Pakistan, we chose the term "partial segregation." Partially segregation of waste includes separating the plastic bottles, dumping the kitchen waste in separate bin, properly decompose the dye and sharps. It takes a value of 1 for those who participate partially in waste segregation activities, and otherwise it takes a value of 0.

5 Health status is another dependent variable Waste related illness – Eight diseases are taken in questionnaire and it takes a value of 1 if the household reports at least one case of any waste-related illness, otherwise 0.

6 HHW generally comprises only 1%–4% of municipal solid waste, but the potential risks to the environment and health are disproportionate to its size. HHW includes RCRA, garden pesticides, mineral oils, heavy metal, Carcinogenic and pharmaceuticals instruments etc.

7 If water Supply lines passing through the drainage takes value of 1 otherwise 0.

8 If using tab water, it takes value 1 otherwise 0.

9 Empty plot where waste is dumping for more than 1 year and in size should be 5 Marla

10 If waste is burnt in the backyard or in empty plot take value 1 otherwise 0.
